# Exposures to Discarded Sulfur Mustard Munitions — Mid-Atlantic and New England States 2004–2012

**Published:** 2013-04-26

**Authors:** Russell Fendick, JC King, Terry Tincher, Marilyn Radke, Gino Begluitti, Miguel Cruz, Mark Keim, Michael Schwartz, Lisa Delaney

**Affiliations:** Non-Stockpile Chemical Material, US Army Chemical Material Activity; Office of the Deputy Assistant Secretary of the Army for Environment Safety and Occupational Health; Chemical Weapons Elimination Program; Office of Environmental Health Emergencies, National Center for Environmental Health/Agency for Toxic Substances and Disease Registry; National Institute for Occupational Safety and Health. CDC

Before the 1970s, the United States sometimes disposed of at sea excess, obsolete, or unserviceable munitions, including chemical munitions (1). Chemical munitions known to have been disposed of at sea included munitions filled with sulfur mustard, a vesicant (i.e., an agent that causes chemical burns or blisters of the skin and mucous membranes) (2). Signs and symptoms of exposure to a mustard agent can include redness and blistering of the skin, eye irritation, rhinorrhea, hoarseness, shortness of breath, and (rarely) diarrhea and abdominal discomfort. Since 2004, CDC has received notification of three separate incidents of exposure to sulfur mustard munitions. In one incident, a munition was found with ocean-dredged marine shells used to pave a driveway. The other two incidents involved commercial clam fishing operations. This report highlights the importance of considering exposure to sulfur mustard in the differential diagnosis of signs and symptoms compatible with exposure to a vesicant agent, especially among persons involved with clam fishing or sea dredging operations.

## Case Reports

### Case 1

In 2004, U.S. Air Force Explosive Ordnance Disposal (EOD) personnel responded to discovery of an artillery shell protruding from a Delaware driveway paved with crushed clamshells (3). They recovered the shell and moved it to Dover Air Force Base for destruction using standard EOD procedures. During handling a “black, tar-like substance” began to drip, and two members required treatment for chemical burns after large pus-filled blisters developed on their hands and arms. One EOD team member required hospitalization as a result of the exposure. Sulfur mustard exposure was confirmed by chemical analysis. After this incident, the Department of Defense made the Army’s policy and procedures for addressing liquid-filled munitions applicable to the Air Force and all other military services.

### Case 2

In 2010, commercial fishermen recovered an unknown number of munitions during dredging for clams off the coast of Long Island, New York (4). During the effort to dump the munitions back in the ocean, a munition was dropped on the deck of the boat, resulting in the release of a black liquid substance. Drops of the substance also landed on the clothing covering the leg and arm of a crew member, and another crew member was exposed to fumes. After several hours, both crew members felt ill and were subsequently transported to a local hospital for evaluation. One crew member was evaluated and released. The other crew member developed small blisters on his forearm and upper thigh. These injuries were recognized by a nurse trained in chemical agent injuries as compatible with exposure to sulfur mustard. Sulfur mustard exposure was confirmed by chemical analysis.

### Case 3

In 2012, a 75-mm projectile was recovered at a clam processing plant in Delaware. Reportedly, it had been brought to the plant accidentally during dredging operations for clams in Delaware Bay. An EOD team removed the munition for disposal (5). The munition involved was determined to contain mustard agent. None of the potentially exposed persons developed signs or symptoms of exposure to mustard. Clam fishermen told investigators that they routinely recover munitions that often “smell like garlic,” a potential indication of the presence of a chemical agent.

## Diagnosis and Management of Suspected Cases

Mustard agent is listed in the Chemical Weapons Convention as an agent used in chemical munitions. Clinicians suspecting mustard exposure should consult with their state or local health department and poison control center regarding the need for follow-up and investigation of potential exposures. CDC’s Chemical Weapons Elimination Program can provide technical consultation and laboratory services to assist clinicians with testing, diagnosis, and management of suspected cases. Program staff members can be contacted through the duty officer at the CDC Emergency Operations Center at 770-488-7100.

Additional information regarding the U.S. Army Chemical Material Activity programs is available by contacting the Public Affairs Office by telephone, 800-488-0648. Additional information regarding CDC programs associated with chemical weapons is available by telephone at 800-CDC-INFO.
